# Are We Transitioning Toward Person-centered Practice on Self-management Support? An Explorative Case Study Among Rheumatology Outpatient Clinic Nurses in Norway

**DOI:** 10.1177/23779608211037494

**Published:** 2021-10-06

**Authors:** Heike Fischer, Kjersti Grønning

**Affiliations:** 1 25574 Trondheim Municipality, Trondheim, Norway; 2 Department of Public Health and Nursing, 8018 Norwegian University of Science and Technology (NTNU), Trondheim, Norway; 3 Department of Rheumatology, St. Olavs hospital, Trondheim University Hospital, Trondheim, Norway

**Keywords:** chronic illnesses, hospital, rheumatology practice, qualitative research, self-management

## Abstract

**Introduction:**

There are only a few studies investigating nurses’ views on self-management in the care of patients with rheumatic diseases.

**Objective:**

The aim of this study is to explore how Norwegian rheumatology outpatient nurses describe their ways of supporting patients’ self-management focusing on the core dimensions of person-centered self-management support.

**Methods:**

Ten individual semistructured interviews with rheumatology outpatient nurses were conducted in Norway from March to September 2017. The interviews were audiorecorded and transcribed verbatim. NVIVO was used to support a systematic analysis of themes and patterns.

**Results:**

Nurses’ views on self-management support fell into three approaches; (1) narrowly biomedically orientated, (2) biomedically and holistic, and (3) person-centered. The nurse's views of self-management support varied and did not fully align with the core dimensions of person-centered practice.

**Conclusion:**

The findings indicate that the biomedical paradigm continues to influence Norwegian rheumatology outpatient clinic nurses’ approach to self-management support. If person-centered principles of self-management support are to be translated into standard nursing practice, including identifying and supporting patient-defined self-management goals and processes, there is a need to challenge established structures in health care systems.

## Introduction

Rheumatic diseases are responsible for a significant part of the disease burden in Norway and globally (Knudsen et al., 2017). While patients with chronic diseases inherently self-manage life with a chronic illness, they may require self-management support from health care professionals at various times (Grønning et al., 2016; Grønning et al., 2017) and according to their individual self-management needs (Sarkar et al., 2008). Patients have distinct self-management support needs and preferences, requiring nurses to offer a range of supportive strategies to support self-management. Research shows that effective self-management support is important as it improves health outcomes (Stenberg et al., 2016) and is crucial to support treatment of patients with chronic diseases (Farley, 2020).

However, an idea around effective self-management support varies for both nurses and patients. Nurses can also experience ethical dilemmas resulting from tensions between beliefs and understandings on the one hand, and practical realities and framing conditions on the other. Despite theoretical insight into what person-centered self-management support entails, nurses may in their practice focus on optimal medical outcomes at the expense of patient autonomy, and may demand active involvement from patients who may either not be willing or not be able to be more actively involved (Dwarswaard & van de Bovenkamp, 2015). At the same time, patients differ in terms of both the importance they place on achieving optimal health, their understanding of optimal health and how to achieve it, as well as how much support they expect in achieving this (van de Bovenkamp & Dwarswaard, 2017).

Person-centered self-management support has been promoted as part of an ideological and sociopolitically driven shift away from producing health for patients to the coproduction of health in collaboration with patients. Person-centered self-management support requires seeing patients as equal partners with expertize about living with a chronic illness with the aim to coproduce health and increase quality of life (Ekman et al., 2011; McCormack et al., 2015; Miles, 2012), while assessing how person-centered self-management support and patient-level outcomes are connected remains a challenge (DeSilva, 2014). Person-centered self-management support has been argued to be better at helping patients to live a good life despite being chronically ill (Dures et al., 2016; Feldthusen et al., 2016; Rathert et al., 2013; Voshaar et al., 2015). In the field of rheumatology, nurse-led care has become a standard model of care in a number of countries including Norway and has been accredited with good clinical patient outcomes (Garner et al., 2017; Bech et al., 2020) and high patient satisfaction (Koksvik et al., 2013) at a potentially lower cost (Larsson et al., 2015). Recommendations by the European League Against Rheumatism for the role of nurses working in rheumatology outpatient care emphasize that a person-centered perspective is important in the care for patients with rheumatic diseases. A person-centered perspective implies that the care is holistic, individually tailored, using a dialog-oriented communication, and collaborative decision making (Bech et al., 2020; Zangi et al., 2015). Although there is no single definition of a person-centered care (Kitson et al., 2013; Rathert et al., 2013), with the terms “person-centered” and “patient-centered” often conflated (Entwistle & Watt, 2013), two core dimensions of person-centered practice are considered central at the level of the clinical encounter. One core dimension is a holistic understanding of the person behind the patient and of her/his individual perspectives, needs, values, expectations, abilities, and capacities. The second dimension is a collaborative relationship between clinician and patient based on mutual respect and understanding, aimed at maintaining and developing patient autonomy (Ekman et al., 2011; Entwistle & Watt, 2013; Dwamena et al., 2012; McCormack et al., 2015; Bech et al., 2020; Voshaar et al., 2015; Zangi et al., 2015). A small number of qualitative studies across a range of both rheumatology and other outpatient settings within Europe have suggested that the biomedical paradigm are dominating in how outpatient clinic nurses support patients’ self-management (Been-Dahmen et al., 2017; Morgan et al., 2017; van Hooft et al., 2015; Wilson et al., 2006). There are only a few studies investigating nurses’ views on self-management in the care of patients with different chronic illnesses (Bos-Touwen et al., 2015; Dwarswaard & van de Bovenkamp, 2015; van Os-Medendorp et al., 2020) or in rheumatology outpatient care (Been-Dahmen et al., 2017). Ethical dilemmas experienced by nurses when providing self-management support always include their beliefs and understandings on “good” self-management and patient autonomy (Dwarswaard & van de Bovenkamp, 2015). It is vital to understand nurses’ understandings of person-centered self-management and the tensions between such understandings and other established ideologies framing nurses’ agency in health care settings. Arguably, a better understanding of person-centered self-management support ideologies is a valuable contribution for understanding how patient participation relates to other values in health care, such as following medical guidelines, ensuring safety, caring for vulnerable patients, and containing health care costs (van de Bovenkamp & Dwarswaard, 2017). To the best of authors’ knowledge, no study of this kind is conducted in rheumatology outpatient care within Norway. Despite international guidelines on what person-centered support entails, nurses may support patients’ self-management in varying ways depending on the cultural, social, and economic context shaping both views and possibilities of action. Such variations may be variations in real and perceived nurse autonomy with professional hierarchies, emphasis on self-reliance, expectations toward nurses’ role in self-management support and so on. Against the backdrop of the core dimensions of person-centered self-management support as outlined above, the aim of this study is to explore Norwegian rheumatology outpatient nurses’ views on self-management support.

## Methods

### Design

A qualitative approach with individual, semistructured interviews was chosen due to the exploratory nature of the research and the need for in-depth knowledge (Bryman, 2016) to answer the research question: How do Norwegian rheumatology outpatient nurses’ express their approaches to self-management support?

### Sample

Nurses working in two different rheumatology outpatient clinics in two Norwegian hospital were invited to participate in this study to gather data on nurses’ self-management support within two different sociocultural contexts. There were three inclusion criteria; the participants had to be fully qualified registered nurses, they had to work in rheumatology outpatient care, and they had to work directly with patients, as opposed to working only in administration or leadership. To achieve symbolic representation (Ritchie, 2014) in terms of gender, years working in rheumatology outpatient care, postgraduate nursing qualifications, and types of consultations (nurse-led consultations or other types), sampling was purposive and convenience based. Using these criteria, 14 nurses were eligible for inclusion. Nurses were informed that participating in the study would be voluntarily, and that their statements would be treated and presented in an anonymized way. Ten nurses agreed to be interviewed and the interviews were conducted between March and September 2017. Both, due to the explorative character of the study and the limited number of rheumatology nurses practicing in Norway, a relatively small sample size were considered suitable (Malterud et al., 2015). The interviews were conducted by the first author, lasted between 45 and 80 min, and took place in an undisturbed environment at the participants’ workplace and during their work time. A semistructured interview guide enabled a systematic exploration of how the nurses described to support patients’ self-management, while facilitating the exploration of potentially unknown issues (Bryman, 2016). The interview guide was based on one used in a study investigating Dutch outpatient nurses’ views on both self-management and their role in supporting self-management (Been-Dahmen et al., 2017) and adapted to the Norwegian context by the authors. The second author is thoroughly familiar with rheumatology services in Norway and the role of outpatient nurses there. The term “self-management” was not translated into Norwegian both for want of a one-word translation and because nurses displayed familiarity with the term during the initial presentations of the project.

### Ethics

This study followed the ethical principles for research involving human subjects (WMA, 2013) and the participants were given both oral and written information about the purpose of the study. Participation was voluntary and no sensitive data were collected. The Norwegian Centre for Research Data approved the study (58522).

### Analysis

The analytical process was a systematic and reflective process where the transferability and trustworthiness of the findings were ongoingly discussed between the authors (Malterud, 2001). Interviews were recorded and transcribed verbatim by the first author, facilitating in-depth, yet systematic analysis (Ritchie, 2014). Overall thoughts and impressions about the interviews were recorded in field notes and employed as an additional guide to inform the final analysis. The interviews were read in their entirety between authors before employing the coding-software NVIVO (QSR, 2017). NVIVO supported a systematic analysis of themes and patterns, facilitating the tracing of analytical processes and iterative cycles (Ritchie, 2014). One example for such iterative cycles was when authors read more work on relational autonomy midway through the analytical process, as this contributed to an understanding of the cardinal distinguishers between views represented in the second and third approaches, respectively. Both authors are experienced in conducting qualitative studies. The first author, who also conducted the interviews and the initial stages of the analysis, was not familiar or had any personal or professional relationships with any of the participants.

## Results

Participants’ characteristics are presented in Table 1, showing that both male and female nurses were interviewed, eight of 10 were nurse specialists, and they carried out different types of consultations, such as independent nurse consultations as part of nurse-led outpatient clinic care, helpline service consultation, and infusion treatment unit consultations. The exact proportion is omitted for anonymity purposes.

**Table 1.** Participant Characteristics.

Sample criteria	Year started to work at the department	Formal nursing qualification	Type of consultations
Participant			
A	1993	NS	INC
B	2012	RN	HS; IT
C	2016	RN	IT
D	1995	NS	INC
E	1999	NS	INC
F	2008	NS	INC
G	2008	NS	IT
H	2009	NS	INC
I	2004	NS	IT
J	2008	NS	INC

*Note*. RN = registered nurse; NS = nurse specialist; INC = independent nurse consultations; HS = helpline service consultation; IT = infusion treatment.

**Figure 1.**
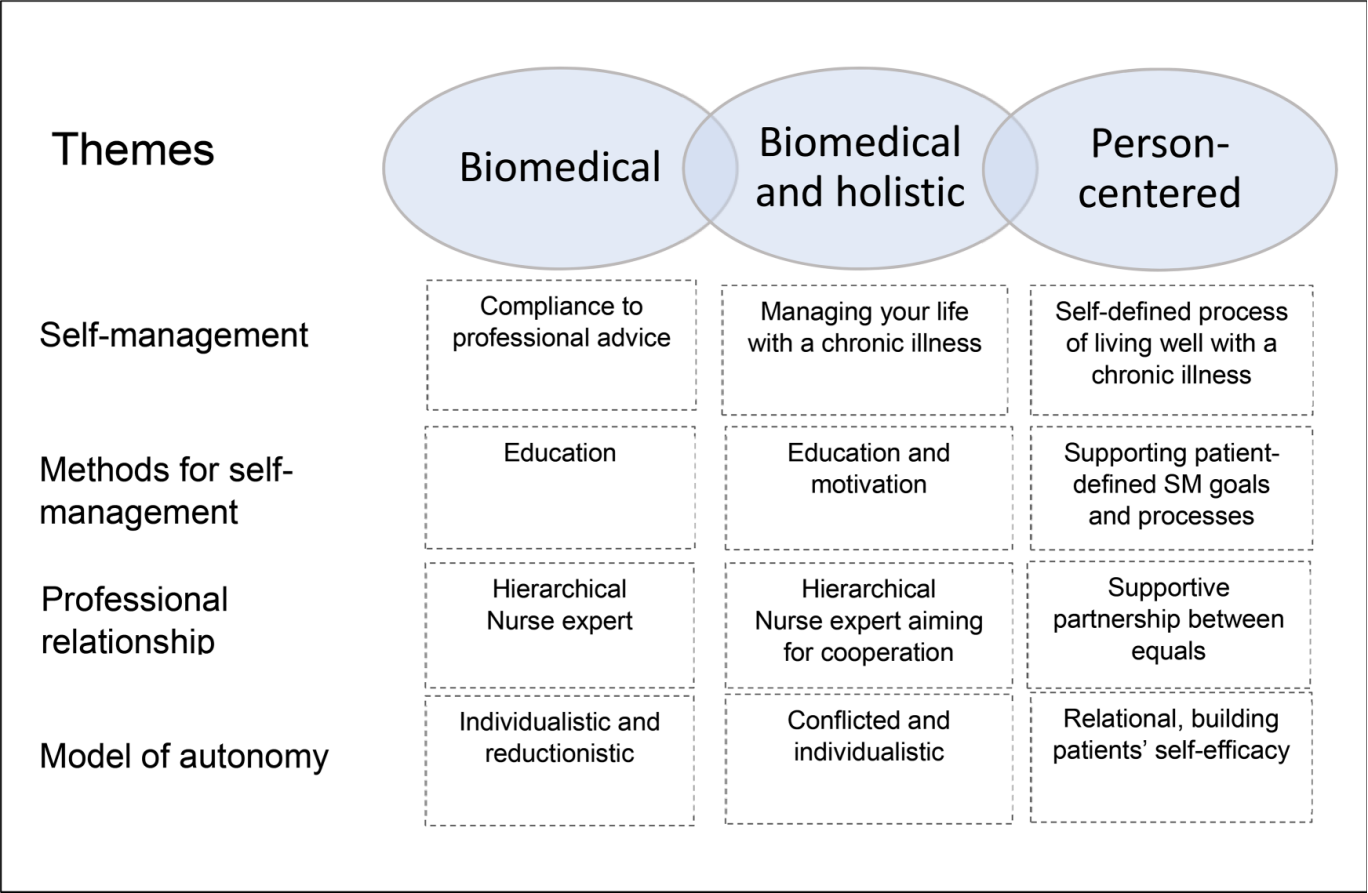
Nurses’ approaches to self-management support.

Our analysis suggests that nurses’ approaches of supporting patients’ self-management ranged from being narrowly biomedically oriented to being person-centered. The approaches were overlapping as illustrated in Figure 1.

In the narrowly biomedically orientated approach, nurses talked about self-management as complying to professional advice and relying on education to provide self-management support. The professional relationship was hierarchical with the nurse as the expert. Trust was described as basis for “good rapport.” In the biomedically but holistically oriented approach, the nurses talked about self-management as managing life with a chronic illness. Their focus was to help patients overcoming issues preventing “good” self-management. In addition to educating patients on ways of self-management from a professional point of view, they focused on developing patient motivation to achieve self-management. The professional relationship was hierarchical while aiming for cooperation, and with nurses emphasizing their role in establishing trust. Trust was considered a basis for “good rapport” where patients’ needs had to be established. In the person-centered approach, the nurses talked about self-management as a self-defined process of living well with a chronic illness. Self-management support had a biopsychosocial perspective, focusing on identifying and supporting patient-defined self-management goals and processes. The nurse–patient relationship was described as a supportive partnership between equals, focusing on building mutual trust, where nurses had an active role in building patients’ self-efficacy. The approaches are further discussed in the following sections.

### The Narrowly Biomedically Orientated Approach

Some nurses proposed an approach to self-management that we interpreted as narrowly biomedically orientated. “Good” self-management was described as a consequence of patients’ understanding of the illness and its treatment. Compliance was described as important in terms of patients adhering to both the medical regimen and lifestyle behaviors recommended by professionals, as well as monitoring symptoms and seeking professional help when necessary. Self-management support was accordingly oriented toward educating patients on issues such as pathophysiology, symptom recognition and management, medications’ side effects, and lifestyle changes. Exemplifying this, one nurse argued that the most important aspect for “good” self-management was that patients adhered to the medical treatment regimen, understood effects and side effects of their medical treatments, and contacted the outpatient clinics when required.

The most important thing regarding self-management is that they take their medication. And that they understand the effects and side effects and contact us when they need to.

(Nurse A)

This perspective was not necessarily responsive to patients’ goals or their varying circumstances and capabilities.

The main focus during consultations tends to be on medical issues. Treatment, side effects, that kind of thing. Although quite often they come with a number of other questions, this and that. But I try to stick to what is to do with the illness.

(Nurse C)

Patients with needs beyond the biomedical regimen were referred to self-management courses or their general practitioners. If education failed to lead to progress toward “good” self-management, nurses repeated or modified their educational efforts. Provided patients were sufficiently informed about “good” self-management practices, and they were considered responsible for being “good” self-managers. The nurse–patient relationship was described in a hierarchical way and with clear boundaries avoiding a partnership. Nurses saw themselves as experts who educate patients in how to manage their illness, its treatment, and the consequences of that. Patients were expected to self-manage as independently as possible and in accordance with professional recommendations.

The word partnership is problematic, because you may get too involved, and help too much. You have to be careful that the patient does not become your partner in a way. There has to be a professional boundary. Our job is to teach patients to manage for themselves and not make them too dependent on us.

(Nurse B)

Although the nurses considered their relationship with patients as important, the relationship was talked about in an instrumental way as to provide “good rapport.” Nurses did not emphasize their role in establishing and maintaining trust and cooperation and relied mainly on more or better information to support patients’ self-management.

We inform them a lot, often repeatedly. But if they don't want to follow our recommendations and get worse… All we can do is to facilitate by informing them well. In the end it is their responsibility. They determine how they want to live their life.

(Nurse A)

### The Biomedically and Holistically Oriented Approach

Some nurses advocated an approach that we interpreted as more holistic and individually tailored type of self-management support than a narrowly biomedically oriented approach.

You need to develop a biopsychosocial understanding of the patient’s situation.

(Nurse F)

Here, self-management was presented as being more than managing the illness and its consequences. Emphasis was placed on patients’ ability to live a “good” life with a chronic illness. Biomedically defined ways of “good” self-management were considered important, yet self-management was also seen to be affected by individual capabilities and life circumstances.

Self-management is, of course, about patients taking their medication to have the best possible health to start with. But it's more than that. You have to learn how to live your life!

(Nurse F)

This approach went beyond educating patients. Nurses’ role was portrayed to be about supporting patients’ identifying and addressing issues concerning intrinsic motivation, self-efficacy, social relations, setting goals and priorities.

The most important thing is that patients take their medication and follow their treatment, but you also need to encourage them to think a little further than just their illness, help them to see what they have both in themselves and around them. That allows them to make the necessary changes.

(Nurse E)

The professional relationship advocated here emphasized that establishing and maintaining trust was important in order to facilitate an ongoing dialogue for patients’ ability to self-manage. Although nurses advocated supporting patients to “work on” a broader range of areas than those advocating a narrowly biomedical approach, the nurse–patient relationship remained hierarchical.

We are not partners, but we have to work as a team and find out about stuff together in order to know what they need most in terms of our support.

(Nurse F)

Self-management support remained conditional to patients aligning their practice to professional understandings of “good” self-management. Thus, while emphasizing a stronger relational focus in self-management support and proposing that patients may require support in negotiating issues affecting self-management, self-management support nevertheless aimed toward professionally defined goals. Interestingly, only nurses proposing this approach expressed frustration or giving up on patients when facing ongoing noncompliance.

It's their life. They need to do it themselves. (…) It can be challenging when patients are dead scared of side effects. (…) I had to say to one patient: “If you don't want the treatment we offer, we are forced to discharge you.” (…) Those kinds of patients challenge me! What happens to your body if you don't get the treatment?

(Nurse D)

### The Person-Centered Approach

Some nurses proposed an approach to self-management that we interpreted as person-centered.

It's about how a patient manages to live life with a chronic illness. And there are many ways of doing that. They need to find their own ways to have the best possible life for themselves.

(Nurse I)

Here, nurses argue for a need to establish an understanding of patients’ individual starting points and focusing on identifying ways to support patients in their self-management that are aligned with patients’ capabilities, motivations, challenges, and importantly, patients’ understandings of both “good health” and a “good life.”

Self-management has to do with the patient's understanding of his health-related challenges and his ability to tackle them so that he can experience health and well-being, whatever that may be.

(Nurse J)

While working against the backdrop of knowledge about biomedically viewed “good” self-management leading to biomedically viewed “good” health, nurses expressed open-mindedness about how self-management may be realized in practice. The goal was not necessarily for patients to become as independent from self-management support as possible, but for patients to be able to live well with their chronic illness.

Much of their ability to self-manage depends on how much else, besides their illness, they may have to deal with. And many have to deal with quite a lot. So, you need to support them holistically and tailored to their situation, because all sorts of things may affect how they are coping.

(Nurse G)

Nurses proposing this approach argued for a nonhierarchical and collaborative nurse–patient relationship, with patients as equal partners assured of professional continued support when needed and/or desired.

I see myself as a helper and not as somebody telling anybody off. It's important that they get the impression that we are there for them no matter what. (…) They may know more about their illness than we do, which is really good, because then they can ask questions. Then we can find out about them and take it from there.

(Nurse I)

The appropriate degree and type of support was seen as dependent on patients’ needs and understandings. Mutual trust between the nurse and patient was considered central in self-management support. Nurses proposing this approach expressed greater awareness of barriers to establishing trust, such as power imbalances and emphasized more strongly their own role in establishing and maintaining trust.

Patients have the lead role. My job is to help them to get behind the steering wheel if they aren't there already and to support them along the way.

(Nurse H)

## Discussion

### Continuing Dominance of the Biomedical Paradigm

Nurses in this study did not consistently describe self-management support in ways that are aligned with the core dimensions of person-centered practice; a subjective and holistic understanding of patients’ self-management, a collaborative partnership between equal partners in care (Ekman et al., 2011; Entwistle & Watt, 2013; Dwamena et al., 2012; Bech et al., 2020; Voshaar et al., 2015; Zangi et al., 2015), and a professional relationship where patients are considered moral agents with the power to define both goals and priorities with regard to self-management support (Entwistle et al., 2018). Nurses in this study described different understandings of “good” self-management support, ranging from narrowly biomedically oriented to person-centered understandings. These findings are in line with other studies finding varying understandings amongst nurses around self-management along the biomedially oriented—person-centered continuum (Dwarswaard & van de Bovenkamp, 2015). While some nurses described more clearly demarcated biomedically oriented understandings and person-centered understandings respectively, others described understandings that incorporated aspects of both biomedically oriented understandings and person-centered understandings of self-management support. Interestingly, it was the latter nurses that problematized nonadherence most strongly arguing this may jeopardize good health outcomes. As discussed later, we suggest this may be a consequence of their underlying understanding of autonomy.

Considering that research suggests that person-centered self-management support is more evident in rhetoric than in practice (Morgan et al., 2017) and may be associated with a “socially desirable response” phenomenon, it can be assumed that responses are skewed toward a person-centered rhetoric. This further strengthens our interpretation of the results not suggesting a successful transition toward person-centered self-management support practices amongst rheumatology outpatient clinic nurses in Norway.

Indeed, nurses in this study considered their practice as person-centered, emphasizing at least elements of person-centered practice. Nurses who propose a type of support that we coined biomedically and holistically oriented aimed to understand patients’ rationales, intrinsic motivations, and subjective experiences of living with a chronic illness, and life circumstances (Berglund et al., 2015; Kendall et al., 2011). Patients’ intrinsic processes are seen to lie behind challenges with their self-management (Dures et al., 2016; van Hooft et al., 2015). The aim is to establish a dialogue creating opportunities for patients to reflect on established understandings, behavioral patterns and goals to promote their “health” (Anderson & Funnell, 2005; Dures et al., 2016). However, nurses with this view contradicted person-centered principles in that the nurse–patient relationship remains hierarchal with nurses being experts who support patients in working toward biomedically defined goals. Nurses with this view saw their role as both educators and motivators with patients needing guidance toward “good” (in a biomedically or professionally defined sense) self-management. As such, patients were not fully acknowledged as moral agents (Entwistle et al., 2018). Self-management is framed by patients’ ideas of a good life, but also influenced by framing conditions outside the patient's domain (van de Bovenkamp & Dwarswaard, 2017). Nurses and patients negotiate self-management on the boundaries between patients’ autonomy and patients’ realities on the one hand and truth around goals and ways of self-management proposed within health care settings on the other hand.

In this study, only nurses proposing a holistically oriented approach expressed frustration when patients over time failed to comply with biomedically defined “good” self-management. A recent study found that nurses’ perception of success may be influenced by how they understand their professional role in self-management support (Duprez et al., 2020). Some nurses kept patients on track and established a rather directive approach while others considered their role as being more comprehensive. The expressed frustration among the nurses in this study can be understood as the result of them on the one hand “investing” more effort into self-management support than nurses arguing for what we coined narrowly biomedically orientated approach, while on the other hand not relenting definitional power of “good” self-management as evident with nurses with a person-centered approach.

### The Need for Reflexivity Around Person-Centered Practice

Others argue that formal education is vital for realizing person-centered self-management support, since nurses need to acquire the necessary knowledge, skills and attitudes to translate theory into practice (Been-Dahmen et al., 2017; Dures et al., 2014, 2016). An interesting reflection is therefore that in this study nurses without postgraduate education where only represented amongst those proposing a narrowly biomedically oriented approach. However, formal education is not sufficient as a cognitive basis for person-centered self-management practice. Nurses operate in environments imbued with implicit and explicit beliefs about what constitutes “good” self-management support practice and need support in developing reflexive understandings about such beliefs in relation to their own practice, as they otherwise may assume to act in a person-centered way when this may not be the case (Ekman et al., 2011; Wilson et al., 2006).

Nurses may also find it difficult to translate person-centered understandings into practice since they experience ethical dilemmas. One such dilemma is patient autonomy versus optimal medical outcomes where patient autonomy may hinder compliance. Others have also found that nurses’ biomedically oriented understandings of self-management frame interpretations of noncompliance as challenging, because in this case noncompliance conflicts with a perception of good self-management (Dwarswaard & van de Bovenkamp, 2015).

Such dilemmas point toward that various discourses and belief systems coexist and need to be negotiated when translating person-centered beliefs into practice. Kendall et al. (2011) argue for example that dominant discourses pertaining both rationalistic, behavioristic assumptions, and an assumed superiority of biomedical knowledge over lay knowledge prevent person-centered self-management support.

These discourses are especially evident amongst nurses proposing a narrowly biomedically orientated approach suggesting that provided patients were informed sufficiently and ought to make the “right” choices. Nurses proposing a biomedically and holistically oriented approach acknowledged both the structural factors that may affect individuals’ varying abilities to self-manage and the need to support patients by strengthening their capabilities. Yet, their understandings of “good” self-management remain biomedically defined. Only some nurses propose ongoing support regardless of patients’ courses of action. This allowed them to acknowledge patients as moral agents while supporting their self-management in a person-centered way (Entwistle et al., 2018). It is ultimately this aspect that makes the kind of support proposed by these nurses person-centered.

### The Centrality of Autonomy and Power

Others have argued for how vital it is to reflect around issues of both autonomy and power to realize person-centered self-management support (Entwistle et al., 2018). Understanding autonomy as a relational concept dissolves the supposed contradiction between patients self-managing on the one hand and receiving ongoing support to self-manage on the other hand (Elissen et al., 2013). Instead, patients and health care professionals can collaborate on defining the goals and priorities for self-management support. Both individual capabilities and autonomy are socially constructed and relationally bound. Being dependent on others’ help does not necessarily contradict autonomy, but can, in fact, promote autonomy and patients’ agency (Entwistle et al., 2018). Nurses proposing a narrowly biomedically orientated approach understood patient autonomy as a choice to not follow professional advice. Empowerment was conceptualized narrowly as enabling patients to appropriately follow professional advice through educating them. This approach fails to acknowledge both the potentially constitutive role of nurse–patient relationships for the development of patient autonomy, and the need to transfer power to the patient in order to promote self-management. In contrast, the conceptualization of autonomy within the biomedically and holistically oriented approach was conflicted. Autonomy was understood as socially constructed and relationally bound, in the sense that patients’ capabilities to self-manage were shaped by various aspects in their lives, including the social relations they were part of. Nurses saw themselves as supporting patients in developing self-management capabilities. Nevertheless, they employed an individualistic understanding of autonomy when rationalizing noncompliance or other ways of “failing” to self-manage according to biomedically defined norms. Self-management support remained thus effectively conditional on patients’ commitment to biomedically defined health goals, thus undermining both patient autonomy and empowerment, and ultimately patients’ capabilities to self-manage with a focus on quality of life (Entwistle et al., 2018). Consequently, nurses found it challenging when patients failed to self-manage according to biomedically defined norms. According to Wilkinson et al. (2016) conflict emerges when nurses meet noncompliance in relation to biomedical norms, while aiming for holistic and person-oriented nursing practice. In contrast, nurses with the narrowly biomedically orientated approach avoided this conflict by employing a rational choice model and by considering their role in self-management support as predominantly educational.

Nurses proposing a person-centered approach did not assume that self-management support needed to be targeted toward biomedically defined goals, thus not experiencing such a conflict. They presented their support as ongoing, holistic, and as a collaboration between equals (Entwistle & Watt, 2013; Entwistle et al., 2018), thus rendering only this approach properly person-centered (Entwistle & Watt, 2013).

### The Need for a System-Level Perspective

Although this study focused on nurses’ views on self-management support instead of structural conditions for self-management support practice, it is important to emphasize that for nurses to translate person-centered theory into practice it is necessary but not sufficient that they are reflexive around their assumptions on self-management support and that they receive appropriate education. Instead, nurses’ self-management support practice is shaped by structural manifestations of an ongoing dominant biomedical paradigm, for example barriers to continuity of care (Berglund et al., 2015), biomedically based definitions of nurses’ roles or tasks (Goh et al., 2006) and limited clinical autonomy (Farrell et al., 2017). These realities may hinder or promote person-centered self-management support (Entwistle et al., 2018; Kendall et al., 2011; Morgan et al., 2017; Wilkinson & Whitehead, 2009; Wilson et al., 2006). For example, Essén and Oborn (2017) found that the use of numerically based treatment progress and targets in a Swedish rheumatology enabled the physicians to identify which patient experiences physicians needed to pay attention to. Physicians’ attention was directed to disease dimensions and predictors while patients wanted to speak about their overall feelings, sleep patterns, or other nonmeasurable dimensions. Arguably, such assessment systems affect over time the prioritizations, attitudes, and understandings of health care personnel. It is in this way that for example treatment targets, assessment systems, or budget decisions may affect cultures and thus nurses’ understandings of self-management support and at the same time their ability to translate their understandings into practice. McCormack et al. 2015 argue that a successful transition toward person-centered practice requires dominant discourses to move beyond person-centered “care” and toward person-centered “cultures.” Person-centered self-management support can only emerge from within person-centered cultures, where nurses can both experience and practice person-centeredness.

### Strengths and Limitations

A strength of this study is that this is, to the best of our knowledge, the first study exploring how Norwegian rheumatology outpatient nurses describe to provide self-management support. Another strength is associated with the researchers’ backgrounds, as they have different academic perspectives and nursing experience. One has a PhD in sociology and works as a nurse in community care nursing; the other has a PhD in nursing, as well as many years of clinical experience from the field of rheumatology nursing. These differences can help prevent that both the research process in general and the analytical process become based around taken for granted disciplinary based assumptions.

A noteworthy limitation is that the study recruited rheumatology nurses from only two outpatient clinics in Norway. Considering that different rheumatology departments in Norway may organize patient care differently, nurses working in other rheumatology departments could have diverging views from those taking part in this study. However, since the sample included nurses with different levels of clinical experience, different formal nursing qualifications and engaged in type of consultations, sufficient heterogeneity of views within the sample is thought to be achieved.

### Implications for Practice

If nurses are expected to realize goals toward person-centered self-management support, they may need support in developing reflexivity on self-management support practice and in addressing structural barriers to being able to practice person-centered self-management support. Structural aspects at various levels of health care systems may also hinder nurses from translating person-centered understandings into practice. In order to translate person-centered self-management support in real practice, nurses and patients should talk about what they consider as good self-management support and the ethical dilemmas that different views may involve (Dwarswaard & van de Bovenkamp, 2015). Discussing ethical dilemmas may contribute to a better understanding about the implications of making different decisions about how patients can self-manage their disease and how the nurses can provide the individual patient with support so the patients values and preference about “what constitutes the good life” is considered.

## Conclusion

Against the backdrop of a broad consensus that a person-centered self-management support practice supports patients with chronic diseases more effectively in their self-management, this study explored how Norwegian rheumatology outpatient nurses describe their approach to self-management support. The findings do not suggest a successful transition toward person-centered self-management support, and the biomedical paradigm instead continues to influence Norwegian rheumatology outpatient clinic nurses’ approach to self-management support. Further research is needed to validate these findings and to develop a better understanding of the underlying factors for diverging views on self-management support. If nurses are expected to realize goals toward person-centered self-management support, they may need support in developing reflexivity on self-management support practice and in addressing structural barriers to being able to practice person-centered self-management support. Structural aspects at various level of health care systems may also hinder nurses from translating person-centered understandings into practice.
